# Femtosecond UV Laser Ablation Characteristics of Polymers Used as the Matrix of Astronautic Composite Material

**DOI:** 10.3390/ma15196771

**Published:** 2022-09-29

**Authors:** Mingyu Lu, Ming Zhang, Kaihu Zhang, Qinggeng Meng, Xueqiang Zhang

**Affiliations:** 1 Beijing Spacecrafts, China Academy of Space Technology, Beijing 100094, China; 2 China Academy of Space Technology, Beijing 100094, China; 3 Laser Micro/Nano-Fabrication Laboratory, School of Mechanical Engineering, Beijing Institute of Technology, Beijing 100081, China

**Keywords:** femtosecond UV laser, resin matrix, bandgap, ablation threshold, energy penetration depth, absorbed energy density

## Abstract

Ultrafast laser processing has recently emerged as a new tool for processing fiber-reinforced polymer (FRP) composites. In the astronautic industry, the modified epoxy resin (named 4211) and the modified cyanate ester resin (known as BS-4) are two of the most widely used polymers for polymer-based composites. To study the removal mechanism and ablation process of different material components during the ultrafast laser processing of FRPs, we isolated the role of the two important polymers from their composites by studying their femtosecond UV laser (260 fs, 343 nm) ablation characteristics for controllable machining and understanding the related mechanisms. Intrinsic properties for the materials’ transmission spectrum, the absorption coefficient and the optical bandgap (*E*_g_), were measured, derived, and compared. Key parameters for controllable laser processing, including the ablation threshold (*F*_th_), energy penetration depth (*δ_eff_*), and absorbed energy density (*E*_abs_) at the ablation threshold, as well as their respective “incubation” effect under multiple pulse excitations, were deduced analytically. The ablation thresholds for the two resins, derived from both the diameter-regression and depth-regression techniques, were compared between resins and between techniques. An optical bandgap of 3.1 eV and 2.8 eV for the 4211 and BS-4 resins, respectively, were obtained. A detectable but insignificant-to-ablation difference in intrinsic properties and ablation characteristics between the two resins was found. A systematic discrepancy, by a factor of 30~50%, between the two techniques for deriving ablation thresholds was shown and discussed. For the 4211 resin ablated by a single UV laser pulse, a *F*_th_ of 0.42 J/cm^2^, a *δ_eff_* of 219 nm, and an *E*_abs_ of 18.4 kJ/cm^3^ was suggested, and they are 0.45 J/cm^2^, 183 nm, and 23.2 kJ/cm^3^, respectively, for the BS-4 resin. The study may shed light on the materials’ UV laser processing, further the theoretical modeling of ultrafast laser ablation, and provide a reference for the femtosecond UV laser processing characteristics of FRPs for the future.

## 1. Introduction

Fiber-reinforced plastics/polymers (FRPs) are comprised of one or more physically or chemically dissimilar elements on microscale fiber phases embedded in a matrix phase [1], which provides many advantages in different areas of engineering and technology [2] and are widely used in the aeronautic and astronautic industry because of their light weight, superior mechanical properties, and inherent corrosion resistance. As a class of two-phase-mixed, anisotropic, inhomogeneous composites, epoxy resin and modified cyanate ester resin are frequently used as the matrix material for the FRP composite, with reinforcing fiber solidified in the resin matrix. For astronautic workpieces made of FRPs, reduced material processing is almost an inevitable process for the final formation of the entire structure. However, the anisotropy and inhomogeneity, as well as the huge mechanical, thermal-dynamical, electrical, and optical properties between the resin matrix and fiber, make it challenging to achieve a uniform removal in the mesoscopical scale, for either traditional machining or electrical machining, or for other nontraditional machining. For example, using traditional mechanical milling, it is easy to produce machining damage (such as edge collapse, fiber damage, and surface tear) and low machining accuracy caused by the huge difference in elasticity and mechanical strength between the resin and fibers, as well the limited removal resolution of the cutting tool’s edge. With the increasing demand for promoting processing accuracy and efficiency and minimizing processing damage, as well as the emergence of new processing requirements (such as fine cutting and surface selective etching of FRPs), the existing contact processing methods have become increasingly difficult, and new processing methods need to be developed [3,4,5].

Laser removal is a non-contact stress-processing method for FRPs, owing to its great advantages over traditional processing methods [6], e.g., it can produce narrower kerfs and higher processing quality than most other processing techniques. Among different lasers, ultrafast femtosecond laser processing serves as a promising machining technique by minimizing thermal damage [7,8,9] and generating high material removal resolution. On the other hand, unlike other lasers, the photon energy of an ultraviolet laser is extremely high, which can destroy the chemical bond in the material molecule, thus directly ionizing it into plasma. Therefore, compared with other lasers, a UV laser usually has stronger material-removal ability, a more extreme processing scale, and less thermal damage when processing materials. It is expected to solve the existing bottleneck processing problems and meet existing and future needs [10]. However, as a result of the huge difference in optical and thermal-dynamical properties between the resin and fiber, there is generally a contrasting laser ablation performance between the two constituents within the FRPs irradiated with ultrafast lasers. For example, by using the intrinsic (namely the near-infrared) femtosecond laser, the resin matrix, which is translucent to the wavelength, usually exhibits a higher ablation threshold than carbon fibers (which is strongly absorbing at the wavelength) at a low number of pulse shots, while the situation reverses when more shots are employed. The former one results in a preferential ablation in carbon fibers beneath or near the resin matrix, while the latter one promotes a selective removal of the resin matrix [11,12]. Consequently, neither of the two cases helps with a fine cutting in FRP sheets (which requires a mesoscopically equal state of removal if the same processing parameters are used). The significant differences in ablation properties for the two constituents, e.g., the ablation threshold and ablation rate, should greatly contribute to the problem. On the other hand, at a low number of pulse shots, the contrast of the removal properties between the resin and carbon fibers is expected to be greatly weakened if a shorter wavelength is used, since resins become much more absorbing while the carbon fibers suffer from minor change in optical response and ablation properties. This makes us believe that an adjustment of the laser wavelength could possibly help with the fine cutting and milling in FRPs, which is of great importance for the small fine-structural processing and high-quality edge cutting used in the astronautic industry.

Here, we prepared a modified epoxy resin (named 4211) and a modified cyanate ester resin (named BS-4), two polymers widely used for the matrix material of astronautic carbon-fiber-reinforced polymer composites in China, according to their material preparation specifications used in the China Academy of Space Technology. The NIR-VIS-NUV liner absorption spectrum was measured to deduce their optical bandgap and for analyzing their femtosecond NUV laser ablation properties. Two of the most important ablation parameters for controllable material processing, namely the energy penetration depth and ablation threshold, were measured and discussed. For the ablation threshold, two commonly used techniques, e.g., the crater diameter-regression method and depth-regression method, were used and compared. A wide range of pulse fluence was considered. The evolution of both energy penetration depth and ablation threshold was discussed analytically. The absorbed-energy densities at the ablation thresholds were also derived analytically. These studies for FRP’s resin matrix may shed light on a fine controllable cutting and milling of FRP material by minimizing the removal property difference with the composites.

## 2. Materials and Methods

### 2.1. Properties of the Samples

As a class, epoxy resin matrix cured at high temperature has excellent comprehensive mechanical properties (high strength and high toughness) [13]. As an upgrade, cyanate ester resin, which has higher temperature resistance, lower moisture absorption, higher toughness, and higher dielectric properties, has become more and more widely used. For astronautic purpose, these pure epoxy resins and cyanate ester resins are usually modified to improve their adaptability to space environments. The modified epoxy resin (named 4211) and modified cyanate ester resin (named BS-4) plates used were specially prepared for astronautic purpose, according to the material preparation specifications used in the China Academy of Space Technology. Specimens were optically polished to 1 mm thickness and were ultrasonically cleaned before the transmittance and ablation experiments. All experiments were carried out at room temperature. The transmittance in 200–1000 nm was obtained by an ultraviolet spectrophotometer (Lambda 1050+ UV-VIS-NIR, PerkinElmer, Waltham, MA, USA). Each piece of data was obtained by averaging over five consecutive measurements.

### 2.2. Laser Ablation and Analysis

**Figure 1:**
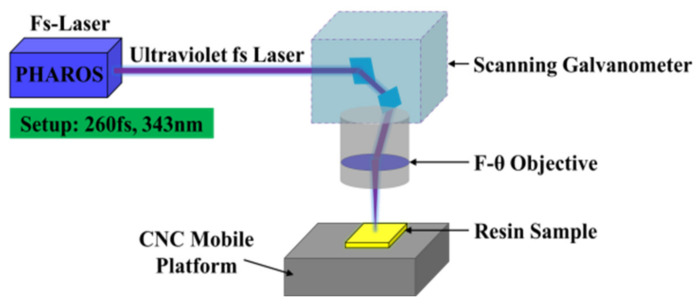
Schematic diagram of femtosecond laser processing system.

Surface ablation experiments were systematically performed on both the 4211 resin and the BS-4 resin plates. Figure 1 shows schematically the diagram of the experimental setup. The femtosecond UV (343 nm) laser (PH1-20 W, PHAROS, Light Conversion, Vilnius, Lithuania, M^2^ ≤ 1.2), generating a linearly polarized, pulsed Gaussian beam, served as the laser source. The temporal size (FWHM pulse duration) of each pulse reaching the target surface was 260 fs. An average power range of 0.05~0.26 W at a fixed pulse repetition frequency of 50 kHz was considered, generating a single pulse energy range of 1 μJ~5.2 μJ. The sample was mounted on a computer-controlled positioning system to provide new material for every shot, if required. A two-dimensional scanning galvanometer and F-theta lens was combined to send focused laser energy onto the samples at almost normal incidence. The number of pulses at one focused spot was adjusted in the range of 1~300 by controlling both the scan speed (with a maximum of 20 m/s) and the number of repeated scans. 

For each resin sample, the on-target Gaussian spot radii *ω*_0_ (1/e^2^ decay of maximum intensity), peak fluences *F*_0_, and ablation thresholds (*F*_th_) were determined, according to the ablated crater diameter-regression technique [14]. The thresholds were also deduced via the crater depth-regression technique [15]. The thresholds deduced by the two techniques were carefully compared. Optical microscopy provided the data for the diameter-regression technique. 3D measuring for ablation craters was based on a laser confocal microscope (LEXT OLS4100, Olympus, Tokyo, Japan), providing the crater ablation depth for the depth-regression technique. Each piece of data for crater diameter or depth was averaged over five consecutive trials.

## 3. Results and Discussion

### 3.1. Transmission and Bandgap Characteristics of Resin Samples

**Figure 2:**
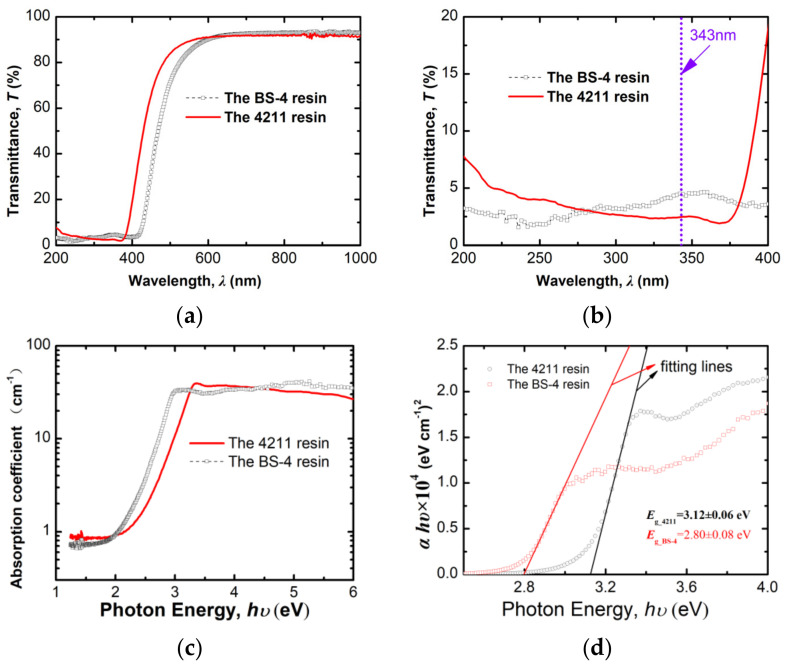
(**a**) Transmission spectrum at 200 nm−1000 nm, (**b**) UV absorption edge, (**c**) Spectrum for absorption coefficient α, (**d**) Relationship between (α*hυ*)^2^ and photon energy, *hυ*.

The transmission (*T*) spectra for the two types of resins are illustrated in Figure 2a,b, with the latter figure showing the details in the UV absorption edges. The absorption coefficient spectra, *α*, derived from the well know relation, *α* = −ln(*T*)/*d*_0_, are shown in Figure 2c, where *hυ* is the photon energy corresponding to the wavelength (*λ*), and *d*_0_ is the sample thickness. Despite the well-known difference in mechanical properties (toughness and breaking strength), the modified epoxy resin (4211) and the modified cyanate ester resin (BS-4) have generally analogous transmittance characteristics in the wavelength range studied. In the UV region studied, the two resins exhibit slightly different optical absorption behaviors; however, they are still comparable to each other, with the absorption coefficient being 30 ± 10 cm^−1^ for the two resins. Particularly, at the laser wavelength (343 nm) used in the following ablation experiments, the transmittance is 2.5% for the 4211 resin and 4.5% for the BS-4, corresponding to an absorption coefficient of 37 cm^−1^ and 31 cm^−1^, respectively.

The direct optical bandgap (*E*_g_) for each resin can be calculated according to the Tauc formula as follows [16]

α*hυ* = A(*hυ* − *E*_g_) (1)

where A is a constant, and *hυ* is the photon energy. By linearly fitting the relationship between (α*hυ*)^2^ and photon energy (*hυ*) at the absorption edges, we yielded the most probable value of 3.1 eV and 2.8 eV for the modified epoxy resin (4211) and the modified cyanate ester resin (BS−4), respectively, differing from each by ~10%. The optical bandgap and absorption coefficient here for the modified epoxy resin (4211), a material particularly fabricated by adding some agent to the pure epoxy resin for astronautic purposes, are still similar to those reported for pure epoxy resin [17], which are 3.45 eV and 40 cm^−1^, respectively. The slight difference between those in [17] and the results here is possibly due to the different subspecies of epoxy resins, as well as to our minor additive to the pure epoxy resin to form the 4211 resin.

### 3.2. Determination of Ablation Characteristic for the Two Resins

The ablation threshold fluence (*F*_th_) is a critical parameter in laser–material interaction for precise control of material processing, which not only governs the lateral size for each laser pulse removal, but also co-determines the vertical size for ultra-short pulsed laser ablation (i.e., ablation rate), together with the physical quantity of effective energy penetration depth (*δ_eff_*) or effective absorption coefficient (α*_eff_*, *δ_eff_* = 1/α*_eff_*). Since the UV (343 nm) laser photon energy (3.6 eV) exceeds both the optical bandgap for the two resins and the laser peak intensity is above ~1 TW/cm^2^, a phenomenal model [18,19] for linear absorption, considering both the one-photon ionization and possibly the avalanche ionization driven by the inverse bremsstrahlung absorption process, can be derived for the experimental determination of the ablation threshold fluence (*F*_th_) and effective absorption coefficient [19], α*_eff_*.

*D*(*F*_0_) = *δ_eff_*ln(*F*_0_/*F*_th_) = α*_eff_*^−1^ ln(*F*_0_/*F*_th_) (2)

where *F*_0_ is the pulse energy fluence, α*_eff_* = α_ion_ + *βE*_g_*n*_e_, with α_ion_ being the one-photon cross-section that originated from the one-photon ionization, and *βE*_g_*n*_e_ originated from the possible avalanche ionization. The relative role of photo-ionization and avalanche ionization for bandgap material was hotly debated in the past two decades (see, e.g., [20,21]). Meanwhile, it is generally accepted that the photo-ionization itself is sufficient to contribute to the reach of ablation for a narrower bandgap material and/or stronger photon energy, in spite of photo-ionization being generally viewed as the seed electrons for avalanche ionization of wide-bandgap material (i.e., avalanche ionization serves as the dominant role of energy deposition [22]). A thorough clarification of these debated problems is beyond the interest of this work. We aim at a phenomenal description of effective energy penetration depth (*δ_eff_*) for guiding the ablation experiments. A depth-regression method based on Equation (1) is used to obtain the ablation threshold fluence (*F*_th_) and effective absorption coefficient (α*_eff_*) for the two resins.

The ablation threshold fluence (*F*_th_) was also derived based the well-known diameter-regression technique [14], i.e., by linearly interpolating the diameter squared to zero, after following the relation, *D*^2^ (*F*_0_) = 2$\omega_{0}^{2}$ ln(*F*_0_/*F*_th_). The ablation threshold and its incubation effect, derived from both the crater diameter-regression depth-regression technique and the depth-regression technique, were compared between types of resin and between techniques.

**Figure 3:**
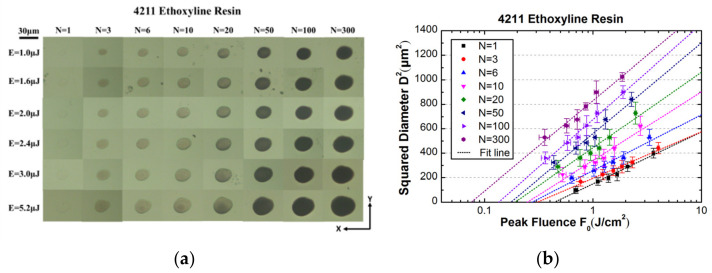
(**a**) Optical microscopy of ablation craters at different laser energies and pulse numbers for the 4211 resin; (**b**) statistics on the crater diameter at different pulse peak fluence for interpolating the ablation thresholds.

The ablation morphology of the epoxy resin at different laser energies and pulse numbers is shown in Figure 3. Seen from left to right, where the incident pulse number is increasing, an incubation effect of ablation threshold is clear. Statistics on the crater diameter based on the diameter-regression technique yielded the value of effective focus diameter for the focused light beam and ablation threshold for the epoxy resin at different numbers of pulses, as shown in Table 1. 

**Figure 4:**
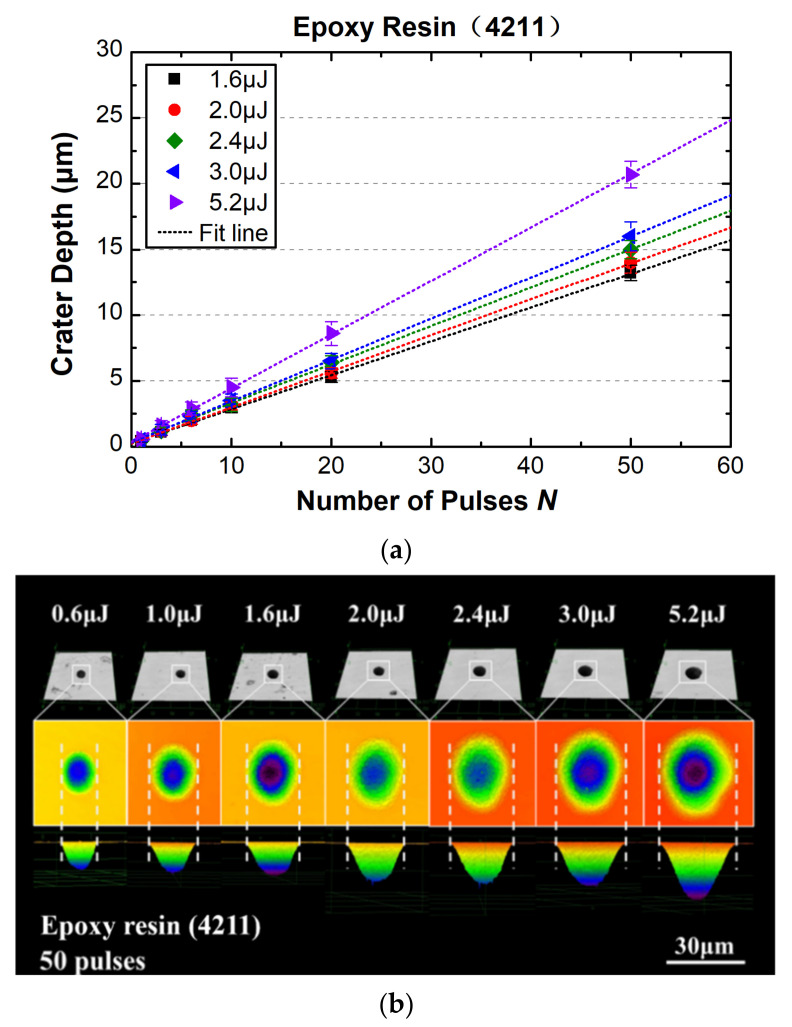
(**a**) Ablation rate with increased pulse number at different pulse energies; (**b**) typical morphologies for 50-pulse ablation of the 4211 resin at different excitations.

Figure 4a shows the plot of crater depth as a function of incident pulse number, at a pulse energy range of 0.6~7.6 μJ, corresponding to the peak fluence ranging from 0.7 J/cm^2^ to about 2.7 J/cm^2^. The well-known phenomenon, i.e., a quasi-linear growth of ablation depth for increasing pulse number, was observed for the epoxy resin, as shown in Figure 4a. A detailed microscopy for the crater’s cross-section showed that a parabolic curve can fit well the cross-section profile for each crater ablated by the single shot (*N* = 1). This is a strong indication that the light absorption process exhibits a linear absorption [23], in view of the spatially Gaussian-distributed energy for the focused beam, which justifies the assumption in Equation (2). Figure 4b shows the evolution of the crater profile at increasing pulse energy, corresponding to 3~26 times the ablation threshold for 50 pulses. The ablation rate, i.e., ablation depth per pulse, keeps increasing with the incident energy. This is in disagreement with the widely reported phenomena for wide-band-gap material irradiated by NIR wavelength, or polymers ablated with a longer wavelength. For example, as reported in [22] for fused silica, reference [24] for PMMA polymer, and reference [25] for various bandgap materials, the ablation rate tends to be saturated from several times the ablation threshold; in other words, a linear absorption law (i.e., a Beer–Lambert behavior) mismatches the ablation behavior at a pulse fluence equal or larger than several times the ablation threshold. 

**Figure 5:**
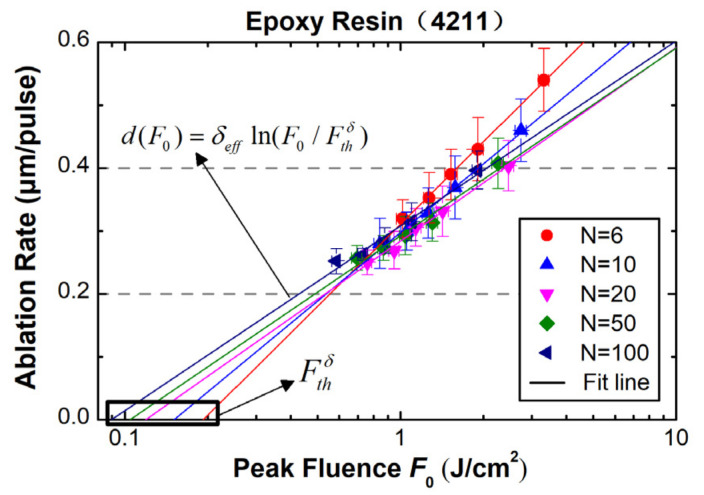
Ablation rate of the 4211 resin as a function of incident laser fluence at different pulse numbers.

Figure 5 shows the logarithm fit of the ablation rate for the 4211 resin ablated with different numbers of pulses ranging from 6 to 100, according to Equation (2). A Beer–Lambert behavior is obvious for all curves. Again, by linearly interpolating the ablation rate to zero, the ablation threshold at different numbers of pulses, as well as the effective energy penetration depth (*δ_eff_*), can be derived as summarized in Table 2.

**Figure 6:**
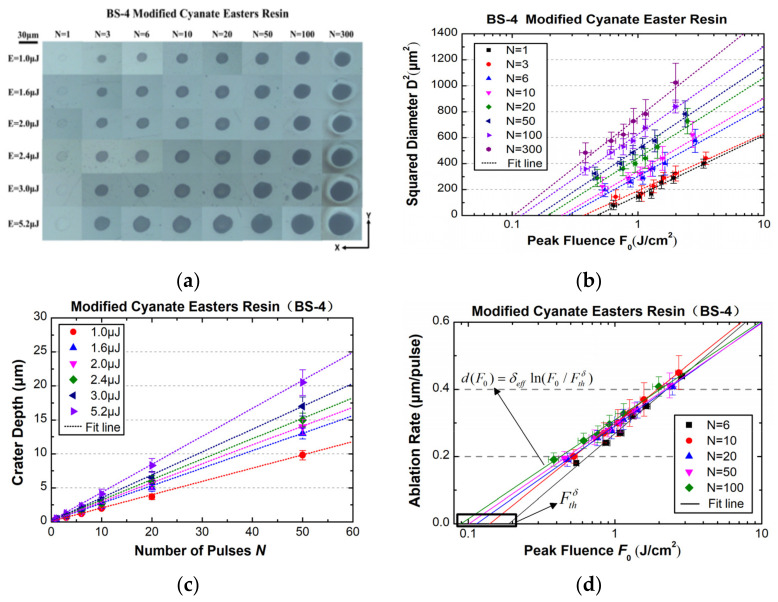
Similar to those in Figure 3, Figure 4 and Figure 5 but for the modified cyanate ester resin (BS-4). (**a**) Optical microscopy of ablation craters; (**b**) statistics on diameter squared at different pulse peak fluences; (**c**) ablation depth as a function of pulse number; and (**d**) logarithm fit of ablation rate.

By following the same procedure, we obtained, respectively, the optical microscopy of ablation craters, diameter-regression-based ablation thresholds, evolutions of ablation rate pulse number, and depth-regression-based thresholds, as shown in Figure 6. In general, similar ablation morphologies with those for the 4211 resin and laws for ablation depth with numbers of pulses were observed. The Beer–Lambert behavior for the ablation rate with pulse fluence is clear, as shown in Figure 6d. The ablation threshold at different numbers of pulses and the effective energy penetration depth of the BS-4 resin can be seen in Table 3.

### 3.3. Comparations of Ablation Characteristics for the Two Resins and Techniques 

**Figure 7:**
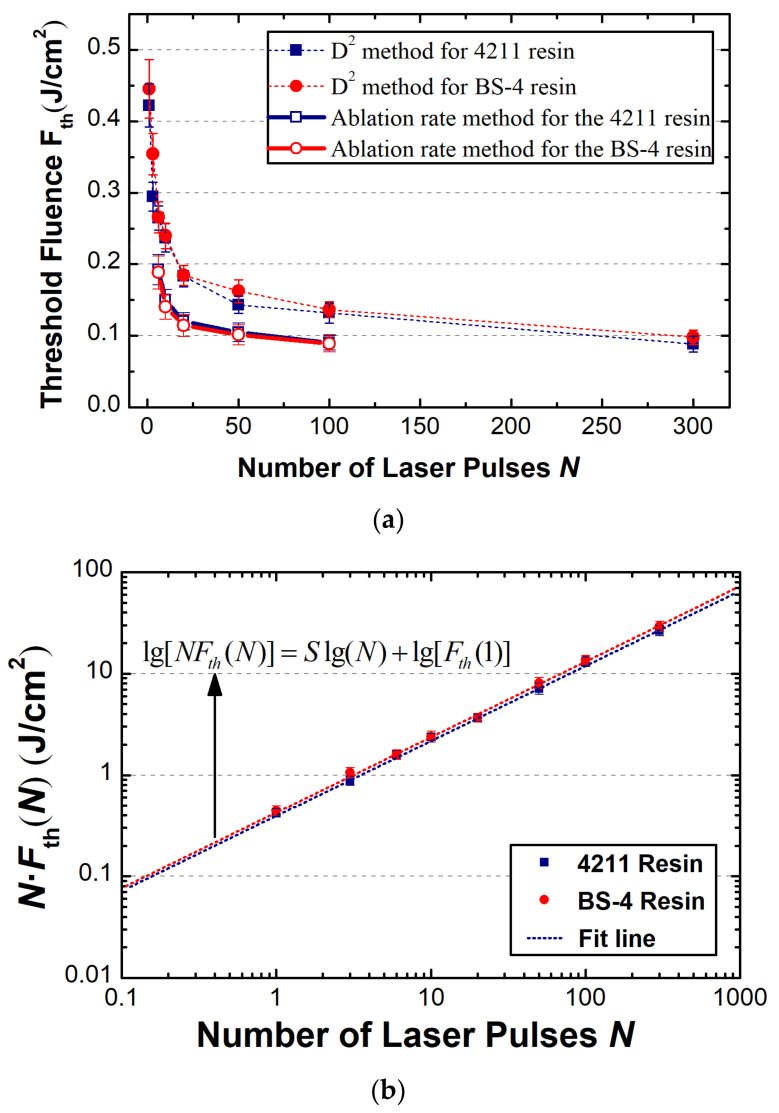
(**a**) Ablation threshold vs. pulse number, namely, incubation behaviors for ablation threshold via two different techniques for both resins; (**b**) accumulated threshold vs. pulse number for deriving the incubation-model-based parameters, where diameter-regression-based thresholds are used.

Solid dots and squares in Figure 7a show the ablation thresholds derived from Figure 3b for the 4211 resin and those from Figure 6b for the BS-4 resin, both of which are based on the diameter-regression techniques. The reduction in the ablation threshold with the number of pulses, namely the phenomenon well-known as the ‘incubation’ effect [26], was reproduced. In general, there is only a negligible difference between the two resins with the experimental uncertainties. The single-pulse ablation thresholds for the epoxy and modified cyanate ester resin are 0.42 ± 0.03 J/cm^2^, and 0.45 ± 0.03 J/cm^2^, respectively. For both materials, the ablation thresholds drop rapidly before they almost saturate at a level of ~0.1 J/cm^2^. This ‘incubation’ effect is caused by the accumulation of defects [26] in the resin induced by multiple laser pulses, causing shrinkage of the effective optical bandgap and, consequently, promoting the optical absorption and reducing the ablation threshold. The saturated ablation threshold at a larger number of pulses is possibly due to the saturated occupation of defects between the valence band and conduction band.

To quantify the incubation effect, we reorganized the data in Figure 7b (with data for single pulse threshold only excluded) by fitting the well-known relation of lg[*NF*_th_(*N*)] = *S*lg(*N*) + lg[*F*_th_(1)] (which is derived from the incubation model *F*_th_(*N*) = *F*_th_(1)*N^S^*^−1^ [15]) to obtain the incubation coefficient (*S*), as well the ablation threshold for a single pulse, *F*_th_(1). The diameter-regression-based thresholds were considered. As expected, the very similarity in thresholds between the two resins yields a close value for both the incubation coefficient and single-pulse threshold, with *S* = 0.73 ± 0.03, *F*_th_(1) = 0.42 ± 0.03 J/cm^2^ for the 4211 resin, and *S* = 0.74 ± 0.02, *F*_th_(1) = 0.45 ± 0.04 J/cm^2^ for the BS-4 resin. We note that the single-pulse threshold for each material derived from the incubation model is quite close to that by the aforementioned measured one. This probably indicates that the aforementioned incubation model fits well to the two resins.

To quantify the difference between the crater diameter-regression technique and depth-regression technique, Figure 7a also demonstrates the ablation threshold from different approaches, with hollow dots and squares representing the depth-regression-based thresholds. The data for the depth-regression technique at larger numbers of pulses (*N* > 100) are absent, considering that reliable information for the ablation rate cannot be obtained in such a situation. Despite the similarities in trend and magnitude, the depth-regression-based threshold is systematically lower than that by the diameter-regression technique by a factor of 30~50%. This discrepancy may be due to the spallation layer that formed in the sub-ablation threshold, which induces an offset in the ablation depth with no steady increase from 0 at the ablation threshold. A similar phenomenon was also recently observed in some metals [27] that exhibited a deviation as large as 50% from those by the diameter-regression technique. Meanwhile, it was shown [27] that the spallation layer played a negligible role in deriving the effective energy penetration depth (*δ_eff_*) in Equation (2). 

**Figure 8:**
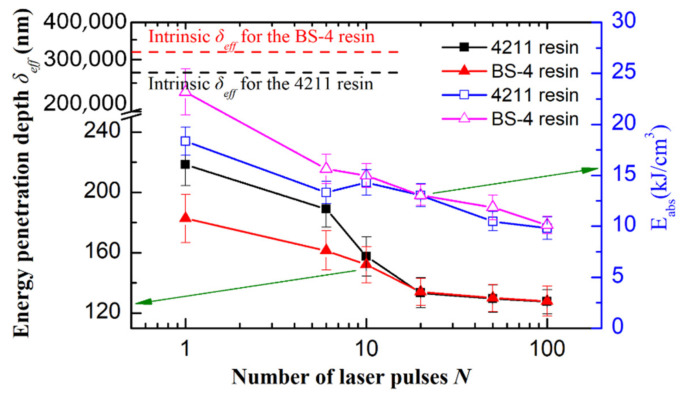
Influence of number of pulses on effective energy penetration depth (*δ_eff_*) and absorbed energy density (*E*_abs_) at the ablation threshold.

Last but not least, to shed some light on a quantitative understanding of the ablation mechanism, Figure 8 compares the evolution of effective energy penetration depth (*δ_eff_*), as well as the absorbed energy density (*E*_abs_) at the ablation threshold, for numbers of pulses (*N*) ranging from 1 to 100. It was found that the initially opaque resins at 343 nm (by weak excitation, as shown in Figure 2b), which have a *δ_eff_* of 250–300 μm, becomes highly absorbing during femtosecond UV (343 nm) laser excitation, reducing the *δ_eff_* by more than three orders of magnitude to 100–200 nm. We observe that the *δ_eff_* shows a similar ‘incubation’ behavior with the ablation threshold shown in Figure 7a. The *δ_eff_* first drops quickly with *N* from *N* = 1 to *N* = 20, followed by a quasi-saturated value of ~130 nm at the larger number of pulses for both resins. We infer that the incubation effect for the *δ_eff_* shares the same physical origin as that of the ablation threshold. In other words, the incubation effect for *δ_eff_* is also probably due to the accumulation of defects in the resin by multiple-laser-pulse irradiation, and the quasi-saturated *δ_eff_* resulted from the saturated occupation of defects between energy bands. We assume a phenomenal expression similar to that for the ablation threshold for the evolution of *δ_eff_* with *N*—for *N* no more than several hundred, *δ_eff_*(*N*) = *δ_eff_*(1)*N^σ^*^−1^. By linearly fitting the relation: lg[*Nδ_eff_*(*N*)] = *σ*lg(*N*) + lg[*δ_eff_*(1)] for the 4211 resin, we obtain an incubation factor *σ* = 0.87 ± 0.03 and *δ_eff_*(1) = 219 ± 13 nm, and they are respectively *σ* = 0.92 ± 0.04 and *δ_eff_*(1) = 183 ± 12 nm for the BS-4 resin. Both linear regression fittings show a correlation coefficient (Adj.R-Square) as high as ~0.99.

Furthermore, we can obtain the absorbed energy density (*E*_abs_) at the ablation threshold for different numbers of pulses, which reads:

(3) $$E_{\mathrm{abs}}(N)=\alpha_{\mathrm{eff}}(N)\times F_{\mathrm{abs}}(N)=\delta_{\mathrm{eff}}^{-1}(N)\times [(1-R)\times F_{\mathrm{th}}(N)]$$

where *F*_abs_(*N*) denotes the absorbed energy fluence, with *R* and *F*_th_(*N*) being, respectively, the reflectivity and incident energy fluence at ablation thresholds (as discussed above). By following the work of Grehn [25] and assuming a close-to-eigenvalue reflectivity [25] (i.e., the Fresnel reflectivity, ~5%), the absorbed energy density (*E*_abs_) at different numbers of pulses was plotted in Figure 8 for both the 4211 resin and BS-4 resin. Equation (3) can also be rewritten as,

*E*_abs_(*N*) ≈ 0.95 × [*F*_th_(1)/*δ_eff_*(1)] × *N^S^*^−*σ*^ (4)

By inserting the empirical expressions for *δ_eff_* (*N*) and *F*_th_(*N*) for the 4211 and BS-4 resins, Equation (4) becomes *E*_abs_(*N*) ≈ 18.4 × *N*^−0.14^ kJ/cm^3^, *E*_abs_(*N*) ≈ 23.2 × *N*^−0.18^ kJ/cm^3^, respectively. In other words, we suggest a maximum absorbed energy density (which is achieved for single-pulse ablation) of 18.4 kJ/cm^3^ and 23.2 kJ/cm^3^, respectively, for the 4211 resin and BS-4 resin to ablation. For modeling, recent work of Grehn [25] suggested intrinsic dissociation energies for predicting the single-pulse-induced crater profile. By following the procedure in [25], taking into account the dissociation energy [28,29,30] for different bonds (e.g., C-C, C=C, and C-F in the two resins), and summing up the bond energies, an dissociation energy (*E*_dis_) around 25 kJ/cm^3^ is obtained for both resins. It is noted that the approximated theoretical value of 25 kJ/cm^3^ is comparable to the aforementioned experimental values, namely, 18.4 kJ/cm^3^ for the 4211 resin and 23.2 kJ/cm^3^ for the BS-4 resin. This indicates that one can employ the bond dissociation energy (*E*_dis_), which is available from reference books (see, e.g., [28,29,30]), as the theoretical absorbed energy density (*E*_abs_) at the single-pulse ablation threshold. Consequently, one can predict the single-pulse ablation threshold (*F*_th_(1)) according to Equation (3), once the effective energy penetration depth (*δ_eff_*) (or effective absorption coefficient, α*_eff_*, *δ_eff_* = 1/α*_eff_*) is obtained through similar experiments or the well-established nonlinear excitation theory (e.g., Keldysh’s theory [31]). In addition, our experimentally determined absorbed energy density (*E*_abs_) at the ablation threshold may shed some light on modeling the ablation process by using the plasma model [18,21], in which a critical energy density is generally required. We note that our single-pulse threshold, *F*_th_(1), and the effective energy penetration depth, *δ_eff_* (1), are in agreement with those in reference [32]. For ablation experiments and related industrial applications, our results suggest that there are only minor differences in ablation characteristics (ablation threshold and its incubation effect, threshold energy density for reaching ablation, etc.) between the 4211 resin and BS-4 resin, and for convenience one does not need to distinguish their carbon-fiber-reinforced composites, if they and their FRP are subjected to ultrafast laser processing. Our recent elaborate design and test of processing FRPs has confirmed this conjecture.

## 4. Conclusions

The work discusses the femtosecond UV laser (343 nm and 260 fs) ablation characteristics of resin matrix for astronautic composite material. From this work, the following conclusions can be drawn.

(1) The transmission spectrum, the absorption coefficient α, and the bandgap of two types of polymer resins were measured and calculated. The bandgap values are 3.1 eV and 2.8 eV for the modified epoxy resin (4211) and the modified cyanate ester resin (BS-4), respectively, which are similar to those of pure epoxy resin reported previously.
(2) The ablation thresholds at 1~300 incident laser shots have been measured for both resins by the diameter-regression technique. The dependence of the ablation threshold on the number of pulses is determined by an incubation effect model. The single-pulse ablation threshold and incubation coefficient for the 4211 resin are 0.42 J/cm^2^ and 0.73, respectively, while they are 0.45 J/cm^2^ and 0.74, respectively, for the BS-4 resin.
(3) The depth-regression-technique-based ablation threshold is systematically lower than that of the diameter-regression technique by a factor of 30%~50%. This phenomenon may result from the spallation-dominated ablation dynamics around the threshold fluence.
(4) The energy penetration depth *δ_eff_* is obtained by the depth-regression technique, and its incubation behavior was modeled similarly to that for the ablation threshold, with an incubation coefficient (*σ*) included. For the 4211 resin, the *σ* and *δ_eff_* for single-pulse excitation are 0.87 and 219 nm, respectively, and they are 0.92 and 183 nm, respectively, for the BS-4 resin. The absorbed energy density *E*_abs_ at the ablation threshold for different numbers of pulses is evaluated analytically, yielding a maximum absorbed energy density of 18.4 kJ/cm^3^ and 23.2 kJ/cm^3^, respectively, for the 4211 resin and BS-4 resin.

## Figures and Tables

**Table 1:** Laser spot diameter and diameter-regression-based ablation threshold for the epoxy resin.

Number of Shot	Spot Diameter 2*ω*_0_ (μm)	Threshold Fluence *F*_th_ (J/cm^2^)
*N* = 1	19.16 ± 0.36	0.42 ± 0.03
*N* = 3	18.17 ± 0.46	0.29 ± 0.02
*N* = 6	19.95 ± 0.41	0.27 ± 0.02
*N* = 10	22.10 ± 0.54	0.24 ± 0.02
*N* = 20	23.18 ± 0.77	0.18 ± 0.02
*N* = 50	24.22 ± 0.72	0.14 ± 0.01
*N* = 100	26.50 ± 0.86	0.13 ± 0.01
*N* = 300	26.69 ± 0.98	0.09 ± 0.01

**Table 2:** Depth-regression-based ablation threshold (*F*_δth_) and effective energy penetration depth (*δ_eff_*) for the 4211 resin.

Number of Shot	*δ_eff_* (nm)	*F*_δth_ (J/cm^2^)
*N* = 6	189 ± 12	0.18 ± 0.03
*N* = 10	157 ± 13	0.14 ± 0.02
*N* = 20	134 ± 10	0.11 ± 0.02
*N* = 50	129 ± 9	0.10 ± 0.01
*N* = 100	128 ± 8	0.09 ± 0.01

**Table 3:** Depth-regression-based ablation threshold (*F*_δth_) and effective energy penetration depth (*δ_eff_*) for the BS-4 resin.

Number of Shot	*δ_eff_* (nm)	*F*_δth_ (J/cm^2^)
*N* = 6	161 ± 13	0.18 ± 0.04
*N* = 10	152 ± 12	0.13 ± 0.03
*N* = 20	134 ± 9	0.11 ± 0.02
*N* = 50	130 ± 9	0.10 ± 0.02
*N* = 100	128 ± 10	0.09 ± 0.01

## Data Availability

The data presented in this study are available in article.
